# Seroprevalence and Associated Risk Factors of *Toxoplasma gondii* in Patients Diagnosed with Schizophrenia: A Case–Control Cross Sectional Study

**DOI:** 10.3390/biomedicines12050998

**Published:** 2024-05-01

**Authors:** Sebastian Grada, Alin Gabriel Mihu, Daniela Adriana Oatis, Constantin Catalin Marc, Liana Maria Chicea, Cristina Petrescu, Alina Maria Lupu, Tudor Rares Olariu

**Affiliations:** 1 Discipline of Parasitology, Department of Infectious Disease, Victor Babes University of Medicine and Pharmacy, 300041 Timisoara, Romania; seba.grada@gmail.com (S.G.); rolariu@umft.ro (T.R.O.); 2 Center for Diagnosis and Study of Parasitic Diseases, “Victor Babes” University of Medicine and Pharmacy, 300041 Timisoara, Romania; marc.catalin@uvvg.ro; 3 “Aurel Ardelean” Institute of Life Sciences, Vasile Goldis Western University of Arad, 86 Rebreanu, 310414 Arad, Romania; danielaoatis@gmail.com; 4 Department of Biology and Life Sciences, Vasile Goldis Western University, 310300 Arad, Romania; 5 Patogen Preventia, 300124 Timisoara, Romania; 6 Department of Infectious Disease, Faculty of Medicine, Vasile Goldis Western University, 310300 Arad, Romania; 7 Department II Medical Clinic, “Victor Papilian” Faculty of Medicine, Lucian Blaga University of Sibiu, 550024 Sibiu, Romania; liana.chicea@gmail.com; 8 Internal Medicine Department, Academic Emergency Hospital, 550245 Sibiu, Romania; 9 Discipline of Hygiene, Department of Microbiology, Victor Babes University of Medicine and Pharmacy, 300041 Timisoara, Romania; petrescu.cristina@umft.ro; 10 Clinical Laboratory, Institute of Cardiovascular Diseases, 300310 Timisoara, Romania; 11 Clinical Laboratory, Municipal Clinical Emergency Teaching Hospital, 300254 Timisoara, Romania

**Keywords:** toxoplasmosis, *Toxoplasma gondii*, schizophrenia, Western Romania

## Abstract

The protozoan parasite, *Toxoplasma gondii*, has been linked to several psychiatric disorders, including schizophrenia. The aim of this study was to assess the prevalence of *T. gondii* IgG antibodies and risk factors associated with seroprevalence in patients diagnosed with schizophrenia. This seroepidemiological study assessed 196 participants, divided into two groups. The study group consisted of 98 schizophrenic patients and was matched with 98 healthy blood donors. A questionnaire was used to gather information regarding potential risk factors associated with T. gondii seroprevalence. Results revealed a higher seroprevalence of *T. gondii* IgG antibodies in schizophrenic patients (69.39%, 68/98) when compared to healthy controls (51.02%, 50/98) (OR: 2.18; 95% CI: 1.21–3.9; *p* = 0.01). Patients with schizophrenia who consumed raw or undercooked meat (80.65%, 25/31) (OR: 3.75; 95% CI: 1.25–11.21, *p* = 0.02) and those with a lower educational level (77.59%, 45/58) (OR: 3.5; 95% CI: 1.59–7.54, *p* = 0.002) presented increased *T. gondii* seropositivity rates versus their control counterparts. Our findings indicate a high *T. gondii* IgG seroprevalence in patients diagnosed with schizophrenia compared to healthy blood donors. Factors associated with *T. gondii* seroprevalence were consumption of raw or uncooked meat and a lower educational attainment. This study provided the first data regarding the potential risk factors for toxoplasmosis in Romanian patients diagnosed with schizophrenia and may serve as a foundation for future research and the development of preventive strategies.

## 1. Introduction

*Toxoplasma gondii*, an intracellular parasite, has been reported to affect about one-third of the world’s population, causing the zoonotic disease known as toxoplasmosis [1,2].

In immunocompetent individuals, acute toxoplasmosis usually presents as a mild or asymptomatic disease [3]. If symptoms are present, they may include cervical posterior adenopathy, myalgia, asthenia, and flu-like symptoms, including fever and mononucleosis-like manifestations [1,4]. Due to the host’s immune response, acute toxoplasmosis progresses into the chronic stage [5]. Chronic toxoplasmosis is characterized by the establishment of latent tissue cysts mostly within skeletal muscles and the brain [6]. The interactions occurring between *T. gondii* cysts and resident cells in the human brain remains poorly understood. Limited data obtained from autopsies indicate the presence of these cysts within neurons and astrocytes [7].

The primary method for diagnosing both acute and chronic toxoplasmosis involves serologic testing. A combination of antibody tests (*T. gondii* IgM, IgA, IgG, and the avidity of IgG antibodies) was employed to distinguish between the acute and chronic phases of infection. The presence of *T. gondii* IgG antibodies, accompanied by high IgG avidity and undetectable IgM and IgA, indicates a chronic infection. The detection of *T. gondii* IgM and IgA antibodies, along with low IgG avidity, is highly suggestive for an acute infection [8,9,10].

Schizophrenia represents a significant global burden of disease, being the 12th leading cause of years lost to disability, placing it ahead of osteoarthritis, chronic obstructive pulmonary disease, and Alzheimer’s disease [11]. Yet, despite significant research, the complex interplay of genetic and environmental factors in its pathogenesis is unclear. Since 1950s, a potential connection between schizophrenia and *T. gondii* infection has been hypothesized [12].

Recent studies reported a significant difference in the prevalence of *T. gondii* IgG antibodies between psychiatric patients with schizophrenia and controls [13,14,15,16]. Infection with *T. gondii* not only increased the susceptibility to schizophrenia but cases with detectable IgG antibodies also exhibited a greater severity when compared to seronegative schizophrenic patients [17,18].

In psychiatric patients, several risk factors were identified to increase the risk of *T. gondii* seropositivity as contact with cats, farming activities that include contact with the soil, consumption of raw vegetables, and raw or undercooked meat consumption [19,20,21].

Limited information regarding the seroepidemiology of toxoplasmosis in Romanian psychiatric patients is currently available. We have previously shown that in patients diagnosed with schizophrenia, the seroprevalence of *T. gondii* IgG antibodies varied from 50.89% in Timis County [22] to 69.77% in Arad County [23]. However, risk factors to *T. gondii* seroprevalence in psychiatric patients were not previously assessed. Therefore, we conducted a seroepidemiological study to evaluate the prevalence of *T. gondii* IgG antibodies and risk factors associated with seroprevalence in patients with schizophrenia from Western Romania.

## 2. Materials and Methods

### 2.1. Study Design

This case–control study was performed between 1 July 2018 and 30 September 2019. We included 196 participants divided into two groups. The study group comprised 98 psychiatric patients who were diagnosed with schizophrenia and admitted to the Psychiatric Clinic of County Emergency Hospital in Arad, Romania. Patients were evaluated by trained psychiatrists in accordance with the Diagnostic and Statistical Manual of Mental Disorders, fifth edition (DSM-V) [24]. Assessment of the medical conditions of study participants was conducted by multiple psychiatrists under the supervision of the chief psychiatrist.

The control group included 98 healthy volunteer blood donors from the Regional Blood Transfusion Centre in Timisoara, Romania. All blood donors had no known psychiatric diagnosis and complied with the strict donation eligibility criteria set by the Romanian Ministry of Health [25]. In order to be eligible for blood donations, participants must present the following criteria: age 18 to 65 years; to not present any fever (fever was defined as a body temperature ≥ 37.5 degrees Celsius) or flu-like symptoms; to not have been diagnosed with chronic diseases (e.g., diabetes, anemia, heart disease, kidney disease, lupus, tuberculosis, hepatitis, epilepsy, syphilis, endocrine disorders, psoriasis, vitiligo, stage III or more obesity, cancer, or head trauma); and to not have undergone any surgical intervention (including tattoos and piercings) within the last 6 months [25]. The control group was matched with the study group by age and gender.

Patients with a history of drug use, individuals whose primary caregiver did not consent to participations, and patients without a caregiver who, due to the nature of their illness, were not able to answer the questionnaire were excluded from this study.

Study participants were grouped according to their age in 5 age categories: 20–29 years, 30–39 years, 40–49 years, 50–59 years, and 60+ years.

### 2.2. Blood Collection and Serologic Tests

Samples were collected upon enrolment in the study for both groups. A standard venipuncture method was used. Blood was drawn into serum separator gel and clot activator tubes (Becton Dickinson, Vaud, Switzerland). Collected samples were then centrifuged at 4000× *g* for 10 min, in 10 to 30 min after collection. The obtained sera were stored into sterile centrifuge Eppendorf tubes and stored at −20 °C until the tests were performed.

Chemiluminescence on Immulite 2000 analyzer (Siemens Healthcare Diagnostics, Malvern, PA, USA) was used to determine *T. gondii* IgG antibodies. All tests were performed in accordance with the manufacturer’s protocol (including quality controls). Results were interpreted based on the manufacturer’s criteria. A value <6.5 IU/mL was considered negative; ≥6.5 IU/mL to 7.99 IU/mL was considered equivocal; ≥8 IU/mL, positive. For the purpose of this study, equivocal results were considered negative.

### 2.3. Questionnaire

Both groups filled out a questionnaire. If the psychiatric patients were unable to complete the questionnaire due to the nature of their illness, the primary caregiver was tasked with completing the questionnaire. In case of the psychiatric patients, the whole processes were conducted by specialized nurses under the supervision of the principal investigators. In case of the blood donors, specialized nurses from the blood collection center were tasked to aid and supervise in the completion of the questionnaire.

The data collection tool consisted of questions regarding the potential risk factors including contact with soil (e.g., farming or gardening), contact with cats (defined as daily contact with felines), contact with dogs (defined as daily interactions with a dog), contact with either a cat or a dog and consumption of raw or undercooked meat, low educational attainment (defined as the participant completing ≤12 grades), and high educational attainment (defined as the participant completing >12 grades).

### 2.4. Statistical Analysis

All the data were collected using Microsoft Excel, version 2011 (Microsoft Corp., Redmond, WA, USA). Categorical data are presented as percentages. Epi Info statistical package 3.3.2 (Centers for Disease Control and Prevention, Atlanta, GA, USA) was used to perform statistical analysis. Mantel–Haenszel chi-square and two-tailed Fisher’s exact tests were used for comparisons between the study group and the control group. Odds ratios (OR) along with their 95% confidence intervals (95% CI) were calculated for each comparison. The *p* values for all hypothesis tests were two-sided. Statistical significance was set at *p* < 0.05.

### 2.5. Ethics and Informed Consent

This study was approved by the Ethics Committee of Emergency County Hospital of Arad, Romania (No. 8051/16 March 2018) and by Victor Babes University Ethics Committee, Timisoara, Romania (No. 05/16 January 2018). A written consent form was signed by all participants.

## 3. Results

The 98 psychiatric patients were aged between 20 and 64 years (mean age = 46.65 ± 9.92 years). The control group comprised 98 donors aged between 20 to 63 years (mean age = 46.37 ± 9.56 years). In both the study and the control group, 44 participants were males (44.89%). In both the study group and the control group, the seroprevalence of *T. gondii* IgG antibodies tended to increase with age (Table 1).

A higher overall seroprevalence of *T. gondii* IgG was found in the study group comprising psychiatric patients when compared to the controls with detectable *T. gondii* IgG antibodies (OR: 2.18; 95% CI: 1.21–3.9; *p* = 0.01).

A significant difference in *T. gondii* seroprevalence was observed between the study group and controls aged 40–49 years: 78.57% (22 out of 28) of psychiatric patients had detectable antibodies, compared to 50% (14 out of 28) rate in blood donors (OR: 3.67; 95% CI: 1.14–11.79; *p* = 0.049).

When the study group aged between 50 and 59 years was compared to controls of the same age, seroprevalence of *T. gondii* IgG antibodies was higher in the psychiatric patients (82.35%, 28/ 34) than in blood donors (48.72%, 19/39) (OR: 4.9; 95% CI: 1.67–14.5; *p* = 0.003).

No difference in prevalence of *T. gondii* IgG antibodies was found in psychiatric patients aged 20–29 years (*p* = 0.44), 30–39 years (OR: 1.2; 95% CI: 0.37–3.92; *p* = 1), and 60+ years (*p* = 0.49) when compared to blood donors of the same age (Table 1).

A significant difference in *T. gondii* seroprevalence was found between psychiatric patients who consumed raw or undercooked meat (80.65%, 25 out of 31) and controls (52.63%, 20 out of 38) (OR: 3.75; 95% CI: 1.25–11.21: *p* = 0.02).

Seroprevalence of *T. gondii* antibodies was higher in psychiatric patients with a low educational attainment (77.59%, 45/58) were compared to their counterparts in the control group (50%, 34/68) (OR: 3.5; 95% CI: 1.59–7.54; *p* = 0.002).

No difference in *T. gondii* IgG seroprevalence was found when the study group who engaged in contact with the soil (OR: 1.34; 95% CI: 0.55–3.29; *p* = 0.65), contact with cats (OR: 0.9; 95% CI: 0.32–2.47; *p* = 1), contact with dogs (OR: 1.43; 95% CI: 0.56–3.68, *p* = 0.47), contact with either cats or dogs (OR: 1.13; 95% CI: 0.49–2.67; *p* = 0.83), and a high educational attainment (OR: 1.18; 95% CI: 0.46–3.07; *p* = 0.8) was compared to the control group (Table 2).

## 4. Discussion

This is the first study that has evaluated the seroprevalence and risk factors of *T. gondii* in psychiatric patients diagnosed with schizophrenia from Western Romania, compared to healthy blood donors. We chose blood donors as controls due to the strict criteria needed to donate blood, meaning that their health condition was as close as possible to “optimal health” [25].

The seroprevalence of *T. gondii* IgG antibodies (69.39%) found in schizophrenic patients was higher than the results reported by Park et al. [26] in Korea (21.9%), Al-Hussainy et al. [27] in Saudi Arabia (31.75%), Cevizci et al. [28] in Turkey (33.3%), Ansari-Lari et al. [29] in Iran (42%), and Campos-Carli et al. [30] in Brazil (56.25%). Similar to our findings, *T. gondii* seroprevalences were reported by Dickerson et al. [31] in USA (64.4%), Alipour et al. [32] in Iran (67.7%), and Hamdani et al. [33] in France (68%). However, a higher *T. gondii* seroprevalence of 74.8% was reported by Esshili et al. [34] in schizophrenic patients from Tunisia. A possible explanation of these differences in seroprevalence could be the difference in culture, socioeconomic status, environmental conditions, number of participants, contact with cats, hygiene, and types of the laboratory test used in the study [20,35].

The current study highlighted a higher prevalence of *T. gondii* IgG antibodies in patients with schizophrenia when compared to the healthy blood donors. Similar results were obtained in several previous published case–control studies conducted on schizophrenic patients in Southern Iran [29], Denmark [36], southeastern China [37], central region of Tunisia [34], upper Egypt [16], Malaysia [14,38], and Lebanon [39]. However, some studies found no association [40,41]. The variability in findings regarding *T. gondii* seropositivity in schizophrenia patients can be attributed to a multitude of factors, including genetic differences, methodological approaches, and the specific psychiatric and cognitive variables measured [39,42,43,44].

In patients suffering from schizophrenia, the presence of an imbalance in the levels of certain neurotransmitters such as dopamine, glutamate, and GABA has been previously reported [45]. This imbalance of dopamine caused by the parasite could contribute to the progression or severity of the disease. The imbalance of dopaminergic pathways, mesolimbic and mesocortical, responsible for motivation, emotional responses, and rewards, has also been found to be involved in the development of schizophrenia [46]. The immune system might also play a significant role in the potential relationship between *T. gondii* and schizophrenia [47]. Chronic infection with *T. gondii* was reported to cause elevated dopamine levels that could lead to the onset of schizophrenia [48]. Other studies have shown that *T. gondii* can produce L-3,4-dihydroxyphenylalanine (L-DOPA) in the brain of rodents infected with this parasite [49]. This has led to the hypothesis that an increase in dopamine level during *T. gondii* infection was associated with behavioral changes [49,50,51]. Treatment with a dopamine inhibitors has been able to alter the behavior of mice infected with *T. gondii* [52]. Brain infection with *T. gondii* affects neuroinflammatory processes, including microglia activation, levels of inflammatory cytokines, and the number of peripheral immune cells that appear during infection. All these biochemical and cellular processes alter behavior in various ways and could affect the risk of developing schizophrenia and the progression of the disease [20,53,54].

Genetic susceptibility represents one of the main risk factors for the development of schizophrenia. It has been reported that individuals infected with *T. gondii* and suffering from schizophrenia have polymorphisms in the genes encoding glucocorticoid-activated kinase 1 (SGK1) and solute carrier family 2 member 12 (SLC2A12), highlighting the potential role of inflammatory processes and infections as risk factors for the development of psychotic behaviors [55]. Infection *with T. gondii* was reported to be a risk factor for the development of schizophrenia in individuals who have this susceptibility or may worsen the disease’s progression, but the parasite alone does not appear to trigger this pathology [5]. The *T. gondii* genome contains two genes that encode the enzyme tyrosine hydroxylase and produce L-DOPA, a precursor of dopamine. The encoded enzymes metabolize both phenylalanine and tyrosine, with a preference for the tyrosine substrate. One of the *T. gondii* genes, TgAaaH1, was constitutively expressed, while the other gene, TgAaaH2, is induced by the formation of bradyzoites during the life cycle of cyst formation, leading to an increase in dopamine levels in the brain, which can produce schizophrenic symptoms [56].

As previously demonstrated [23], we found a higher seroprevalence of *T. gondii* IgG antibodies in females diagnosed with schizophrenia when compared to blood donor females. No significant differences in the seroprevalence of *T. gondii* IgG antibodies was noted between schizophrenic patients and those with healthy blood who acknowledged contact with soil. This aligns with findings published by Teimouri et al. (2022) in Fars Province, Southern Iran, who also reported no difference in seroprevalence linked to soil contact in psychiatric patients versus controls [19]. Our results are in contrast with Cong et al. (2015) who identified a positive association in Eastern China [57]. A potential explanation for these discrepancies could be the varying climate conditions across study locations. While *T. gondii* oocysts can remain infectious for up to 18 months under ideal conditions [58], their survival rate is reportedly higher in humid tropical climates compared to arid or cold regions [19,59,60], possibly influencing seroprevalence rates.

Our results did not find an association between detectable *T. gondii* IgG antibodies and contact with cats when schizophrenic patients were compared to blood donors. Similar results were obtained by Elsaid et al. (2014) when comparing the seroprevalence of *T. gondii* IgG antibodies of psychiatric patients to random volunteers in Libya [61]. A possible explanation is that the risk of infection from cats is limited to the brief period when they shed *T. gondii* oocysts, typically not more than 21 days [62,63]. Moreover, if cats are kept indoors and are not fed raw meat, they do not come into contact with this parasite [64].

In this study, the presence of *T. gondii* IgG antibodies in schizophrenic patients were associated with the consumption of undercooked or raw meat. Similar results were obtained by Cong et al. (2015) when comparing psychiatric patients to a control group comprising the general population from Eastern China [57]. Previous reports suggested that humans can come into contact with this parasite through the ingestion of raw or undercooked meat contaminated with tissue cysts of a infected intermediate host [62,65,66]. Pigs, poultry, sheep, and goats are considered important intermediate hosts for *T. gondii* [67]. A possible explanation for this association is the high consumption of organic meat in Western Romania [68]. The likelihood of infection with *T. gondii* in organic meat appears to be higher than conventionally reared animals due to the continuous potential exposure to contaminated soil [64]. Another explanation could be the consumption of processed meats, especially pork, traditional for Western Romania [69]. If the curing process is not prolonged enough (more than 12 months), Herrero et al. (2017) reported up to 153 parasites per gram of dry-cured ham [70]. Previous studies conducted on blood donors [71] and pregnant females [72] found no direct link between meat consumption and *T. gondii* infection. However, Olariu et al. (2020) reported that pregnant women working with meat were at risk of exposure to *T. gondii* through the ingestion of raw or undercooked meat while tasting dishes [72]. This risk of exposure to *T. gondii* through routinely tasting dishes was underscored by reports from U.S. studies indicating that only one in three women were aware of the potential *T. gondii* contamination from consuming raw or undercooked meat [72,73,74].

A higher difference of *T. gondii* IgG antibodies was found in schizophrenic patients with a lower education attainment when compared to healthy blood donors. Similar results were observed by Sirin et al. in bipolar patients [75]. Lower education attainment was associated with a decreased understanding and awareness of *T. gondii* infection and its preventative measure, increasing the likelihood of exposure [71,76].

We did not find any difference in *T. gondii* seroprevalence when comparing patients with schizophrenia with healthy blood donors who come into contact with dogs. Dogs could play a role in the transmission of *T. gondii* by contaminating their fur with oocysts. In addition, if a dog feeds on contaminated cat feces, it can defecate oocysts [71,77,78]. Our results align with those obtained by Alvarado-Esquivel et al. when comparing patients suffering from mental and behavioral disorders due to psychoactive substance use to controls from the general population.

Our study acknowledges certain limitations. One is the small sample size, including study participants aged 20–29 years and 60+ years. The blood donor group comprised healthy individuals aged between 18 and 65 years and may not reflect the seroprevalence of *T. gondii* antibodies in the general population, due to the stringent health criteria required for blood donation [71]. This could contribute to a selection bias, as these individuals might have lower overall infection rates of any kind. It is possible that this could have led to an overestimation of the association between *T. gondii* and schizophrenia. The potential correlation between schizophrenia, age, and the fact that blood donors are eligible for donation within a limited age range should be also considered [79,80,81]. The diagnosis of schizophrenia was not further categorized into specific subgroups (as paranoid, catatonic, undifferentiated, etc.) and we did not investigate the course of the disease. In addition, we used a questionnaire that did not include factors as living conditions, socioeconomic status, educational status, or detailed dietary habits. Also, cross-sectional studies identify associations, not a temporal relationship, complicating the determination of whether exposure preceded the observed outcome or not. Participant’s recall accuracy can introduce measurement error. Due to their single-time-point nature, cross-sectional studies are limited in their ability to establish causation or sequence events and primarily serve to reveal associations [82]. Another limitation of our study was that we did not control for the increase in familywise error rates throughout the statistical analysis. We considered this research preliminary and encourage replication.

## 5. Conclusions

Results of the present study indicate that *T. gondii* IgG seroprevalence was higher in patients diagnosed with schizophrenia compared to healthy blood donors. A higher seroprevalence rate was also noted in female schizophrenic patients, in psychiatric patients who engaged in the consumption of raw or uncooked meat, and in patients with lower educational attainment when compared to the control group. Our study brings important and new data regarding the seroprevalence, and the potential risk factors associated with *T. gondii* infection in patients diagnosed with schizophrenia. Further seroepidemiological studies in Western Romania should be considered to better understand the relationship and potential risk factors between *T. gondii* infection and schizophrenia.

## Figures and Tables

**Table 1:** Seroprevalence of *T. gondii* IgG antibodies in the study and control groups, according to age group and sex.

	Number of Participants with Detectable *T. gondii* Antibodies/ Total Number of Participants Tested (%)		OR	95% CI	*p* Value
Age Group	Study Group (*n* = 98)	Control Group (*n* = 98)			
20–29	0/5 (0)	2/5 (40)	N/A.		0.44
30–39	12/22 (54.55)	11/22 (50)	1.2	0.37–3.92	1
40–49	22/28 (78.57)	14/28 (50)	3.67	1.14–11.79	**0.049**
50–59	28/34 (82.35)	19/39 (48.72)	4.9	1.67–14.5	**0.003**
60+	6/9 (66.67)	4/4 (100)	N/A.		0.49
**Sex**					
Female	41/54 (75.93)	30/54 (55.56)	2.52	1.11–5.75	**0.04**
Male	27/44 (61.36)	20/44 (45.45)	1.91	0.82–4.45	0.2
Total	68/98 (69.39)	50/98 (51.02)	2.18	1.21–3.9	**0.01**

*n* = number of participants; N/A. = not applicable; ***p* value < 0.05**.

**Table 2:** Potential risk factors for *T. gondii* infection in the study group and control group.

Risk Factor *	Number of Participants with Detectable *T. gondii* Antibodies/ Total Participants Tested (%)		OR	95% CI	*p* Value
	Study Group (*n* = 98)	Control Group (*n* = 98)			
Contact with soil	30/46 (65.22)	21/36 (58.33)	1.34	0.55–3.29	0.65
Contact with cats	52/79 (65.82)	15/22 (68.18)	0.9	0.32–2.47	1
Contact with dogs	39/54 (72.22)	20/31 (64.52)	1.43	0.56–3.68	0.47
Contact with either cats or dogs	63/92 (68.48)	21/32 (65.63)	1.13	0.49–2.67	0.83
Consumption of raw/uncooked meat	25/31 (80.65)	20/38 (52.63)	3.75	1.25–11.21	0.02
Low educational attainment	45/58 (77.59)	34/68 (50)	3.5	1.59–7.54	0.002
High educational attainment	23/40 (57.5)	16/30 (53.33)	1.18	0.46–3.07	0.8

* = Participants who answered “Yes” were included in statistical analysis; ***p* value < 0.05**.

## Data Availability

Data are available upon request.
